# Chemical Constituents Analysis and Antidiabetic Activity Validation of Four Fern Species from Taiwan

**DOI:** 10.3390/ijms16022497

**Published:** 2015-01-22

**Authors:** Chen-Yu Chen, Fu-Yu Chiu, Yenshou Lin, Wei-Jan Huang, Po-Shiuan Hsieh, Feng-Lin Hsu

**Affiliations:** 1 College of Pharmacy, School of Pharmacy, Taipei Medical University, 250 Wuxing St., Taipei 110, Taiwan; E-Mails: d301097006@tmu.edu.tw (C.-Y.C.); wjhuang@tmu.edu.tw (W.-J.H.); 2 Graduate Institute of Pharmacognosy, School of Pharmacy, Taipei Medical University, 250 Wuxing St., Taipei 110, Taiwan; 3 Department of Life Science, National Taiwan Normal University, No. 162, Sec. 1, Heping E. Rd., Taipei 106, Taiwan; E-Mails: richisland0418@gmail.com (F.-Y.C.); yenshoulin@ntnu.edu.tw (Y.L.); 4 Department of Physiology and Biophysics, National Defense Medical Center, No. 161, Sec. 6, Minquan E. Rd., Taipei 114, Taiwan; E-Mail: pshsieh@mail.ndmctsgh.edu.tw

**Keywords:** pterosin, reactive oxygen species (ROS), rat pancreatic insulin-secreting (RINm5F) cells

## Abstract

Pterosins are abundant in ferns, and pterosin A was considered a novel activator of adenosine monophosphate-activated protein kinase, which is crucial for regulating blood glucose homeostasis. However, the distribution of pterosins in different species of ferns from various places in Taiwan is currently unclear. To address this question, the distribution of pterosins, glucose-uptake efficiency, and protective effects of pterosin A on β-cells were examined. Our results showed that three novel compounds, 13-chloro-spelosin 3-*O*-β-d-glucopyranoside (**1**), (3*R*)-Pterosin D 3-*O*-β-d-(3'-*p*-coumaroyl)-glucopyranoside (**2**), and (2*R*,3*R*)-Pterosin L 3-*O*-β-d-(3'-*p*-coumaroyl)-glucopyranoside (**3**), were isolated for the first time from four fern species (*Ceratopteris thalictroides*, *Hypolepis punctata*, *Nephrolepis multiflora*, and *Pteridium revolutum*) along with 27 known compounds. We also examined the distribution of these pterosin compounds in the mentioned fern species (except *N. multiflora*). Although all pterosin analogs exhibited the same effects in glucose uptake assays, pterosin A prevented cell death and reduced reactive oxygen species (ROS) production. This paper is the first report to provide new insights into the distribution of pterosins in ferns from Taiwan. The potential anti-diabetic activity of these novel phytocompounds warrants further functional studies.

## 1. Introduction

Ferns are a group of approximately 12,000 species belonging to the botanical group known as Pteridophyta. Certain fern species are consumed as food or as folk medicine in several countries to treat various ailments. Ferns primarily contain flavonoids, alkaloids, phenols, steroids, and triterpenoids; exhibit various bioactivities such as antibacterial, antiosteoporosis, and anti-Alzheimer’s disease activity; and possess hypolipidemic and hypoglycemic activities [1]. Therefore, ferns are a major medicinal resource in ethnopharmacy.

Pterosin, sesquiterpenes with 1-indanone skeletons, were first isolated from the bracken fern *Pteridium aquilinum* var. *latiusculum* (Pteridaceae) [2]. Approximately 31 pterosins have been isolated from several fern species (Table S1) and exhibit anticancer, smooth-muscle relaxation, and leishmanicidal activities [3]. Pterosin A was expressed against type 1 and type 2 diabetes in an animal model. In addition, further research has indicated that pterosin A can promote glucose uptake, improve insulin sensitivity, and enhance adenosine monophosphate-activated protein kinase (AMPK) phosphorylation, which regulates carbohydrate and fatty acid metabolisms [4]. Therefore, pterosin compounds may be useful for treating metabolic disease in future studies.

Oxidative stress damages several cellular functions in the pathophysiology of various diseases. Reportedly, reactive oxygen species (ROS) were produced by macrophages and were responsible for apoptosis or necrosis of insulin-secreting cells [5]. β-Cell compensation for insulin resistance occurs by increased insulin secretion or cell mass, and lack of compensation causes glucose intolerance [6]. ROS production has been associated with β-cell dysfunction and cell death in both type 1 and type 2 diabetes [7]. Chronic exposure to long-chain saturated fatty acids is another major inducer of type 2 diabetes. Accelerated free fatty acid (FFA) production will promote oxidative process in mitochondria, which may also enhance ROS production. Moreover, with an irregular protein synthesis rate, the endoplasmic reticulum accumulates with increasing unfolded protein levels in the lumen, which is associated with abnormal oxidation. Aggregated misfolding proteins may cause excess ROS production, inducing gradual apoptosis of pancreatic β-cells [8].

AMPK is a cellular sensor that regulates energy and metabolic homeostasis; it activates in response to increased ratio of AMP to adenosine triphosphate and calcium ion content. AMPK is a master regulator in the physiology of several organs, regulating carbohydrate, lipid, and protein metabolism. AMPK activity primarily maintains the glucose content within the physiological range in various cells, particularly β-cells [9]. However, increased AMPK activity can suppress insulin secretion to prevent exhausted β-cells [10]. Impaired functional β-cell production after chronic compensation reduces insulin secretion and AMPK activation, which may potentiate glycolipotoxicity-induced cell death [11]. Therefore, the AMPK pathway is crucial for regulating glucose homeostasis and is a major target of therapy for type 2 diabetes.

However, the actual distribution and content of pterosin analogues in certain ferns from Taiwan remains unclear. In the present study, we isolated 30 phytochemicals from four fern species: *Hypolepis punctata* (Thumb.) Mett, *Ceratopteris thalictroides* (L.) Brongn, *Nephrolepis multiflora* (Roxb.) Jarret ex Morton and *Pteridium revolutum* (BI.) Nakai. Among these, 13-chloro-spelosin 3-*O*-β-d-glucopyranoside (**1**), (3*R*)-pterosin D 3-*O*-β-d-(3'-*p*-coumaroyl)-glucopyranoside (**2**), and (2*R*,3*R*)-pterosin L 3-*O*-β-d-(3'-*p*-coumaroyl)-glucopyranoside (**3**) are novel compounds. Here we describe the structural elucidations of **1**, **2**, and **3**. These pterosin compounds were evaluated for their antidiabetic activity. In addition, we developed and validated a sensitive and specific method involving liquid chromatography-tandem mass spectrometry (LC–MS–MS) for analysis of these pterosins.

## 2. Results

### 2.1. Structural Elucidation

**Figure 1:**
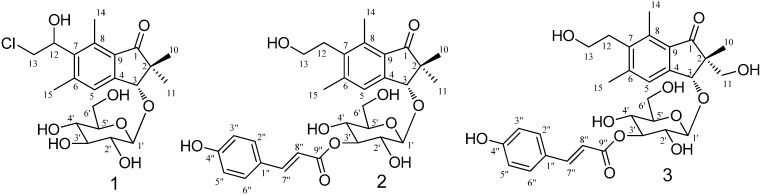
Structures of Compounds **1**–**3**.

Fresh fern material from *H. punctata*, *C. thalictroides*, *N. multiflora*, and *P. revolutum* was extracted using organic solvent. Repeated chromatography on silica gel and highly porous polymer gel produced three new compounds (Figure 1) in addition to 27 known compounds, which were determined by comparing their physicochemical and spectroscopic data with published reports.

Compound **1** was obtained as a colorless oil. The IR spectra at 1598 and 1697 cm^−1^ indicated the presence of a benzene ring and carbonyl group. Characteristic ^1^H-NMR spectra revealed signals assignable to gem-dimethyl (δ 1.07, 1.61 (each 3H, s, H-10, 11)), two aromatic methyl groups at δ 2.50 (3H, s, H-15) and 2.73 (3H, s, H-14), one chloroethyl group (δ 3.93 (2H, m, H-13), 5.40 (1H, dd, *J* = 5.4, 5.2 Hz, H-12)), one allylic oxygenated methylene at δ 4.84 (1H, s, H-3), and one aromatic proton (δ 7.53 (1H, s, H-5)). In addition, the ^1^H-NMR shifts at δ 3.27–4.56 suggested one sugar moiety. These signals indicated the presence of a pteroside skeleton. On the basis of the correlation spectroscopy (COSY) and heteronuclear multiple quantum coherence (HMQC) spectra, the glycosidic moieties were assigned as a glucopyranose. The configuration of the anomeric position (δ 4.56) was confirmed as a β-configuration by the coupling constant (*J* = 7.7 Hz). The heteronuclear multiple bond coherence (HMBC) correlations between glucopyranose H-1' and aglycone C-3 suggested that glucose was substituted at C-3. Moreover, ESI-MS revealed isotopic [M + H]^+^ ion peaks at *m*/*z* 443/445, and the molecular formula of Compound **1** was suggested as C_21_H_29_ClO_8_. A comparison of this aglycone with spelosin [12] revealed an upfield shift of the C-13 spectra; thus, the chlorine group was attached at C-13. Acid hydrolysis of **1** gave the aglycone and glucopyranose, rescpectively, and their structures were confirmed by comparison of the ^13^C-NMR spectra with those of references. The absolute configuration of aglycone was determined by the specific rotation with a value of [α]_D_^24^ + 82.6 (*c* = 0.7, MeOH) similar to that of spelosin ([α]_D_^22^ + 83.3 (*c* = 0.7, MeOH)) [12]. Consequently, Compound **1** was determined as 13-chloro-spelosin 3-*O*-β-d-glucopyranoside.

**Figure 2:**
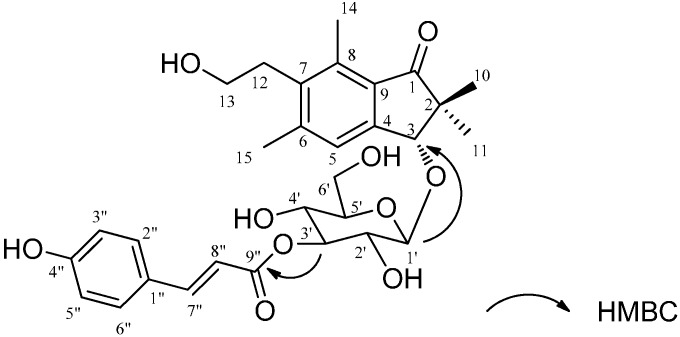
HMBC (heteronuclear multiple bond coherence) correlations for Compound **2**.

The molecular formula of Compound **2** was C_30_H_36_O_10_Na, as determined from HR-ESI-MS *m*/*z* 556.2312 [M + Na]^+^. The ^1^H-NMR showed gem-dimethyl at δ 1.08 (3H, s, H-10), 1.29 (3H, s, H-11), two aromatic methyl groups at δ 2.46 (3H, s, H-15) and 2.63 (3H, s, H-14), two coupled methylenes of a hydroxyethyl group (δ 3.30 (2H, t, *J* = 7.7 Hz, H-12) and 3.60 (2H, t, *J* = 7.7 Hz, H-13)), one allylic oxygenated methylene at δ 4.85 (1H, s, H-3), and one aromatic protons (δ 7.57 (1H, s, H-5)) for a pterosin D skeleton, along with a *p*-coumaroyl group (δ 6.40 (1H, d, *J* = 15.8 Hz), δ 7.66 (1H, d, *J* = 15.8 Hz), δ 6.80 (2H, d, *J* = 8.4 Hz), and δ 7.46 (2H, d, *J* = 8.4 Hz)), except for the presence of sugar signals. According to the COSY and HMQC spectra, the glycosidic moiety was assigned as a glucopyranose. The HMBC correlation between glucopyranose H-1' and aglycone C-3 suggested that glucose was substituted at C-3 of pterosin D. A comparison of the ^1^H-NMR spectra for Compound **2** with (3*R*)-pterosin D 3-*O*-β-d-glucopyranoside revealed a downfield shift of H-3' (δ 5.12) of the glucose moiety, which supported together with the HMBC signal H-3'/C-9" the linkage of the *p*-coumaroyl group to C-3' (Figure 2). Comparison of the specific rotation of pterosin D ([α]_D_^24^ + 4.8 (*c* = 0.5, MeOH)), which was obtained by acid hydrolysis of **2**, with that of previously isolated (3*R*)-pterosin D ([α]_D_^22^ + 5 (*c* = 0.35, MeOH)) [12] led to the (3*R*)-configuration of **2**. Additionally, based on the result of NOESY correlation of H-10/H-3 and H-11/H-1', the absolute configuration of **2** was suggested to be the same as that of (3*R*)-pterosin D. Accordingly, Compound **2** was identified as (3*R*)-pterosin D 3-*O*-β-d-(3'-*p*-coumaroyl)-glucopyranoside.

The molecular formula of Compound **3** was determined as C_30_H_36_O_11_Na *m*/*z* 572.2263 [M + Na]^+^ by HR-ESI-MS. The ^1^H- and ^13^C-NMR data (Table 1) were similar to those of Compound **2**, except for the one hydroxymethyl group at H-2 of the 1-indanone skeleton. Compound **3** revealed a *p*-coumaroyl moiety, a glucopyranose unit, and pterosin L as determined by the NMR data [12]. From the HMBC data, the correlation of H-3 with anomeric carbon (C-1') suggested that glucose was substituted at C-3 of pterosin L. In addition, the substantial downfield shift of H-3' indicated the connection site of the coumaroyl group. The HMBC correlation demonstrated that the H-3' linkage was located at the conjugated carbonyl of *p*-coumaroyl. Acid hydrolysis of **3** yielded pterosin L, *p*-coumaric acid, and glucopyranose. The optical rotation of pterosin L with a value of [α]_D_^24^ + 19.5 (*c* = 1.1, MeOH) were consistent with literature values ([α]_D_^22^ + 20 (*c* = 0.25, MeOH)) [12]. Thus, Compound **3** was structurally elucidated as (2*R*,3*R*)-pterosin L 3-*O*-β-d-(3'-*p*-coumaroyl)-glucopyranoside.

**Table 1:** ^1^H- and ^13^C-NMR spectra for compounds **1**, **2** and **3**.

Position	1		2		3	
	δ_H_ (*J* in Hz)	δ_C_	δ_H_ (*J* in Hz)	δ_C_	δ_H_ (*J* in Hz)	δ_C_
1		211.1		211.3		207.9
2		52.8		52.6		55.9
3	4.84, s	86.0	4.85, s	86.5	4.74, s	84.1
4		153.1		151.8		145.2
5	7.53, s	128.0	7.57, s	126.8	7.54, s	125.0
6		146.0		146.2		136.5
7		140.2		138.3		132.3
8		139.0		138.6		137.2
9		131.5		130.9		131.3
10	1.07, s	22.7	1.08, s	22.0	1.22, s	17.2
11	1.61, s	22.2	1.29, s	22.8	3.56, m	65.7
12	5.40, dd (5,4, 5.2)	71.9	3.30, t (7.7)	33.1	3.00, t (7.7)	31.7
13	3.93, m	47.8	3.60, t (7.7)	61.6	3.58, t (7.7)	60.2
14	2.73, s	15.1	2.63, s	14.1	2.65, s	12.7
15	2.50, s	22.0	2.46, s	21.4	2.47, s	20.0
1'	4.56, d (7.7)	105.9	4.70, d (7.7)	105.8	4.64, d (7.9)	104.3
2'	3.27–3.43, m	75.3	3.00–4.00, m	73.8	3.00–4.00, m	72.2
3'	3.27–3.43, m	78.2	3.00–4.00, m	79.1	3.00–4.00, m	77.3
4'	3.27–3.43, m	71.7	3.00–4.00, m	69.9	3.00–4.00, m	68.4
5'	3.27–3.43, m	78.0	3.00–4.00, m	77.9	3.00–4.00, m	76.7
6'	3.72–3.75, m	62.9	3.72–3.93, m	62.5	3.70–3.80, m	60.9
1"				127.3		125.9
2",6"			7.46, d (8.4)	131.1	7.50, d (8.6)	129.7
3",5"			7.64, d (8.4)	116.8	6.80, d (8.6)	115.4
4"				161.2		159.9
7"			7.66, d (15.8)	146.6	7.67, d (16.0)	144.9
8"			6.40, d (15.8)	115.6	6.41, d (16.0)	114.1
9"				169.1		167.6

### 2.2. LC-MS-MS of Pterosins A, I, and Z

**Figure 3:**
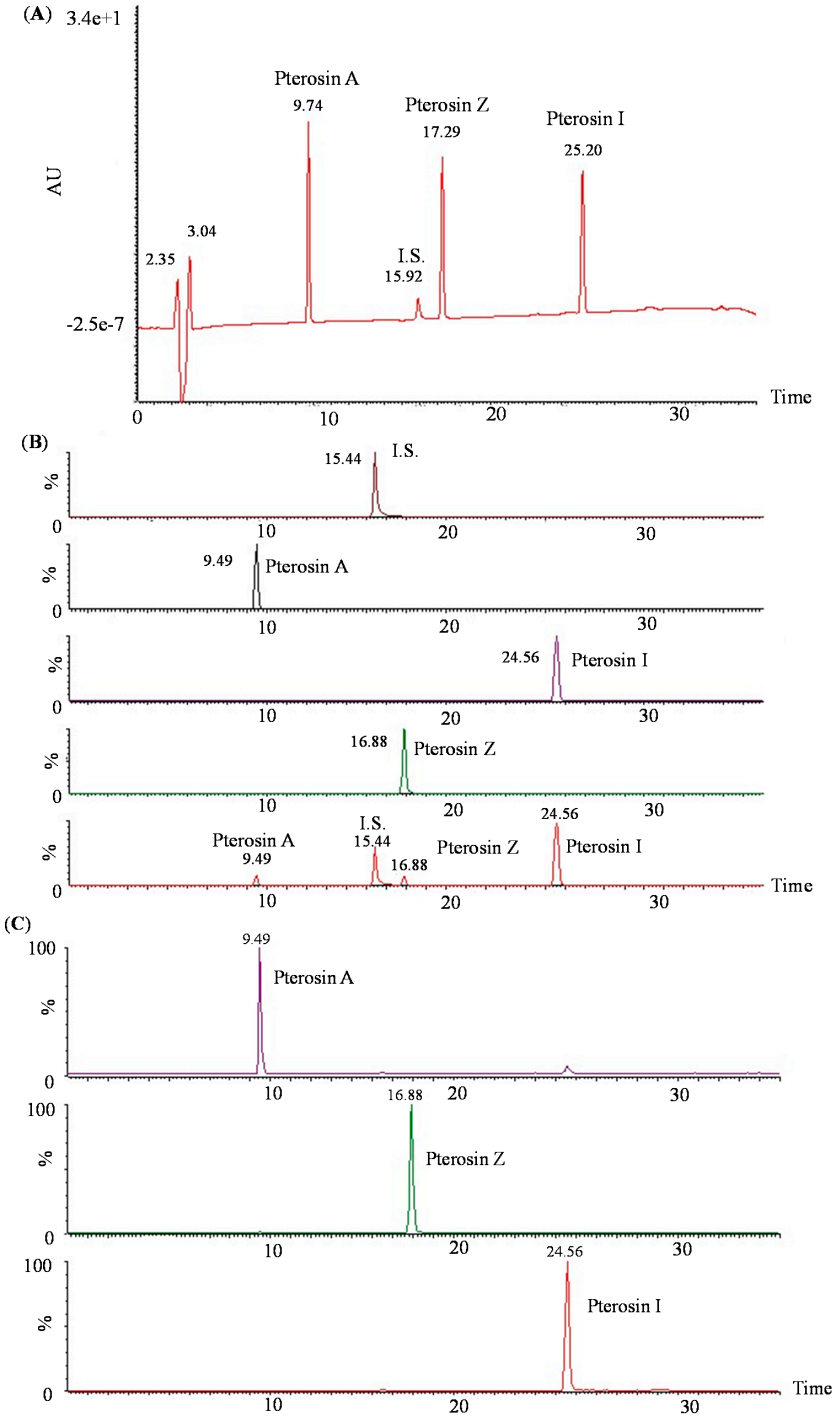
LC–MS–MS chromatography of pterosins. (**A**) High-performance liquid chromatography of pterosins A, I, and Z and piromidic acid (internal standard); (**B**) Multiple reaction monitoring chromatography corresponding to the LC–MS–MS analyses of pterosins and piromidic acid and (**C**) daughter ion chromatograms.

We analyzed the isolated pterosins by LC–MS–MS. Figure 3 presents the MRM and daughter ion chromatograms obtained for analyzing the pterosin mixture of the analytes.

**Figure 4:**
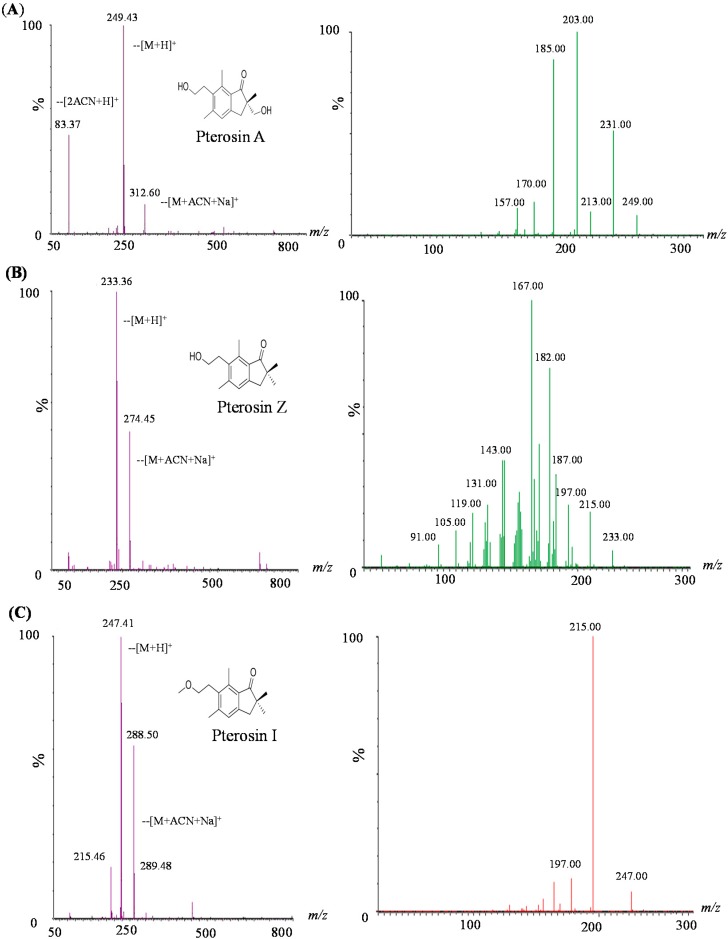
First mass scan analysis and daughter ion mass spectra of three pterosins: (**A**) pterosin A; (**B**) pterosin Z; and (**C**) pterosin I.

Fragmentation patterns of the precursor ions were observed for pterosins (A, Z, and I) when these were analyzed using ESI with a triple quadrupole MS. After CID, the [M + H]^+^ of the aforementioned pterosins produced a major fragment ion at *m*/*z* 249.43, 233.36, and 247.41, respectively. Each [M + H]^+^ pterosin of the parent ion was screened based on the first paragraph. The cleavage fragments (daughter ions) were detected by a second mass analysis. Pterosins of daughter ion mass spectra revealed collision energies of 18 eV (pterosin A and I) and 28 eV (pterosin Z) (Figure 4). Each of the three components exhibited fractured fragments, and the relative strength of the various peaks of fragments can be used to identify the features of the constituents.

### 2.3. Biology Activity

#### 2.3.1. Pterosins Increased Cellular Uptake of Glucose

**Figure 5:**
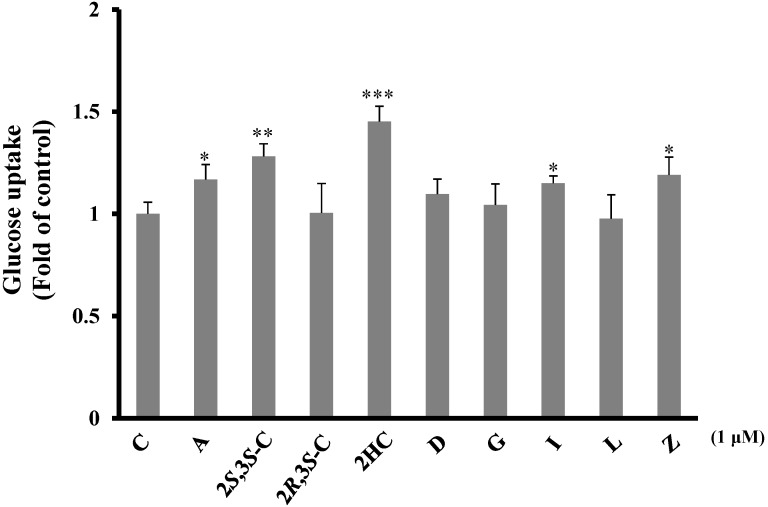
Effects of the isolated pterosins on glucose uptake in C2C12 myocytes. The cells were treated with the test compounds (1 μM) and 2-deoxy-d-[^3^H] glucose was added to determine the glucose uptake activity. * *p* < 0.05, ** *p* < 0.005, *** *p* < 0.001.

We investigated the glucose uptake activities of pterosins in C2C12 myocytes based on the 2-deoxyglucose uptake levels after a 20-min treatment with 1 µM of the aforementioned pterosin compounds. 2-Hydroxypterosin C and (2*S*,3*S*)-pterosin C significantly increased glucose uptake (*p* < 0.01), as indicated by the mild elevation with pterosins A, I, and Z (*p* < 0.05) (Figure 5).

#### 2.3.2. Pterosin A Protected H_2_O_2_-induced Reactive Oxygen Species (ROS) through Adenosine Monophosphate-Activated Protein Kinase (AMPK) Activation

**Figure 6:**
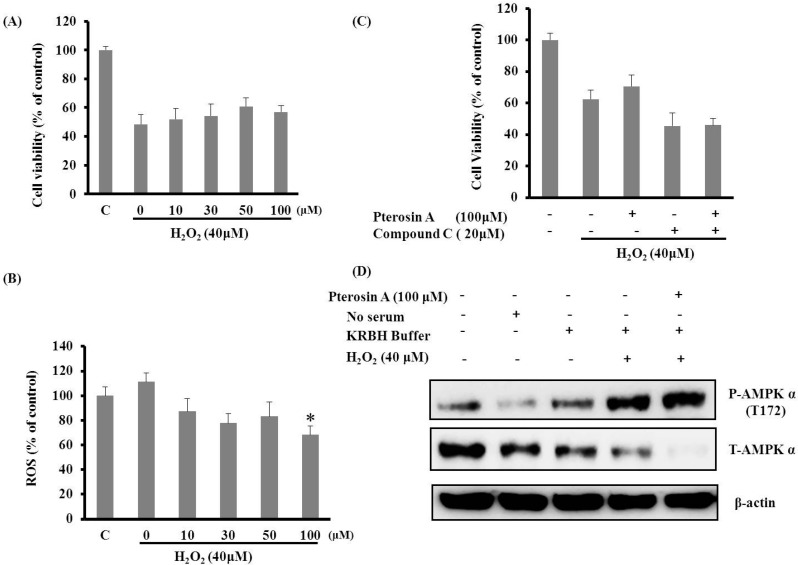
Pterosin A protected against H_2_O_2_-induced reactive oxygen species (ROS) cell damage through adenosine monophosphate-activated protein kinase (AMPK) activation. (**A**) RINm5f cells were treated with 40 μM H_2_O_2_ for 2 h and then incubated with various doses of pterosin A for 18 h; (**B**) The cells were coincubated with H_2_O_2_ (40 μM) and various doses of pterosin A for 18 h. The ROS levels were determined using an NBT assay; (**C**) Cell viability on incubation with H_2_O_2_ with and without pterosin A (100 μM) and Compound **C** (AMPK inhibitor) for 2 h; (**D**) The cells were incubated with H_2_O_2_ for 2 h and exposed to pterosin A, followed by western blot analysis of phospho-T172 AMPK and total AMPK levels. Data are presented as mean ± SEM. * *p* < 0.05.

The generation of ROS, including hydroxyl radicals (·OH), H_2_O_2_, and superoxide anion (O_2_^−^), and the concomitant formation of NO was associated with β-cell dysfunction and cell death [7]. The RINm5f β-cells were incubated with various concentrations of pteroisn A with and without 40 μM H_2_O_2_; subsequently, the cell viability and ROS levels were determined using MTT and NBT assays, respectively. Pterosin A exhibited a mild protective effect through H_2_O_2_-induced cell death, and the scavenging capacity effect of ROS was dose-dependent (Figure 6A,B); therefore, pterosin A may, as an antioxidant, reduce oxidative stress-induced cell death in β-cells.

Pterosin A was found to be a novel AMPK activator. In addition, AMPK phosphorylation inhibits NO-induced apoptosis [13]. Therefore, we examined the protective effects of pterosin A on cells through AMPK activation. The AMPK activation was more substantial with H_2_O_2_ pretreatment than that with pterosin A or H_2_O_2_ alone (Figure 6C); however, Compound **C** attenuated the protective effects of pterosin A on H_2_O_2_-induced oxidative stress (Figure 6D). Thus, the cytoprotective effects of pterosin A might be partially mediated through AMPK activation.

#### 2.3.3. AMPK Activation Avoided Palmitate-Induced Lipotoxicity by Pterosin A

**Figure 7:**
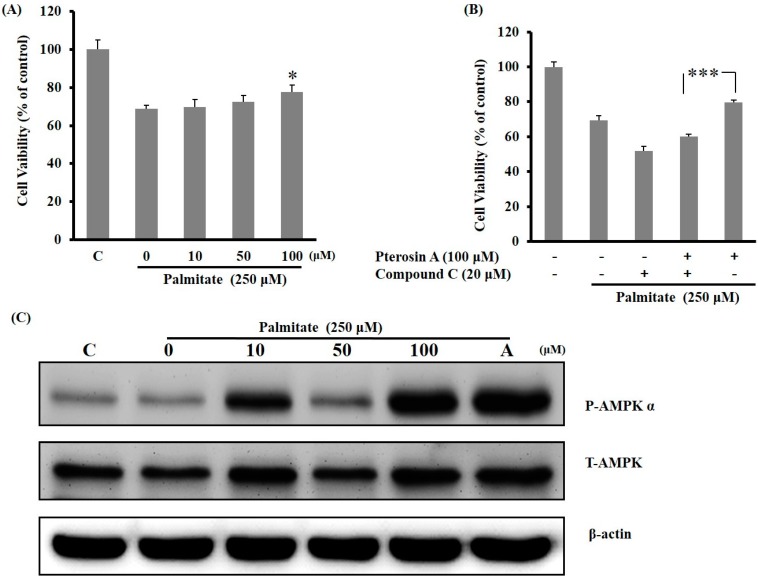
Effects of pterosin A on palmitate-induced lipotoxicity and AMPK expression in RINm5F cells. (**A**) Cell viability on cotreatment with various doses of pterosin A and palmitate (250 μM) for 24 h; (**B**) The cells were pretreated with Compound **C** (20 μM) for 2 h, followed by cotreatment with plamitate (250 μM) with and without pterosin A for 24 h; (**C**) Western blot analysis of total and phospho-AMPK (A: 5-aminoimidazole-4-carboxamide ribonucleotide (AICAR) was used as a positive control). Data are presented as mean ± SEM. * *p* < 0.05; *** *p* < 0.001.

H_2_O_2_ is produced by oxidative stress, which may result from excess glucose or lipid intake. In the present study, the RINm5f β-cells were pretreated with Compound **C** before incubation with palmitate and pterosin A cotreatment. Cell viability decreased with antioxidant palmitate, and palmitate with Compound **C** also reduced cell viability, but this diminished cell viability was dose-dependently reversed by pterosin A (Figure 7A,B). Pterosin A dose-dependently enhanced the AMPK phosphorylation in the palmitate-stimulated β-cells by at least 24 h (Figure 7C). Therefore, pterosin A might play a protective role in reducing lipotoxicity-induced cell death in β-cells through AMPK activation.

#### 2.3.4. Pterosin A Inhibition in Palmitate-Induced ROS Production

**Figure 8:**
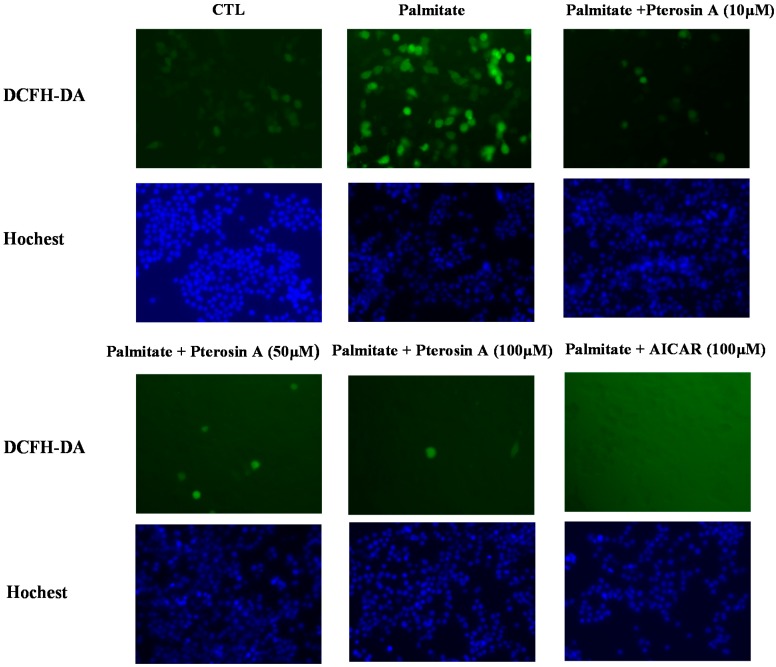
Effects of pterosin A on palmitate-induced ROS in RINm5F cells. The cells were exposed to various doses of pterosin A with palmitate (250 μM) (A: AICAR was used as a positive control). Fluorescent microscopy to determine the ROS levels by 2',7'-dichlorofluorescein diacetate (DCFH-DA) staining (original magnification 200×), Hoechst staining of nuclei.

A recent study indicated that inhibition of ROS plays a protective role in palmitate-induced β-cell apoptosis [14]. We assessed ROS generation by 2',7'-dichlorofluorescein diacetate (DCFH-DA) staining in β-cells. The palmitate-treated RINm5f cells revealed increased ROS levels at 24 h (Figure 8). Moreover, pterosin A revealed a dose-dependent reduction in ROS production.

## 3. Discussion

Pterosins comprise a large group of sesquiterpenes, and these compounds occur widely in the Dennstaediaceae and Pteridaceae families. We isolated the seasonal variations of pterosins compounds and other components from four fern species, including nine pterosins, five pterosides, six lignans, three flavonoids, six phenolics, and one carbohydrate, along with photochemicals from *C. thalictroides* and *N. multiflora*. In addition, Compounds **21** to **23** were identified for the first time in *H. punctata*. Moreover, seven compounds (Compounds **10** to **12**, **14**, and **25** to **27**) were identified in *P. revolutum* for the first time. Furthermore, the results revealed that the distributions of the pterosin compounds and pterosin A in the three aforementioned species (*H. punctata*, *C. thalictroide*, and *P. revolutum*), except *N. multiflora* (Nephrolepdiaceae), were higher than the corresponding distributions of the other pterosin analogs (Table S2). Several previous studies have isolated several triterpenes and steroids from Nephrolepdiaceae [15]. These findings clearly indicated the presence of nonpterosin-type components in *N. multiflora*.

However, whether pterosin A has protective effects on pancreatic β-cells against oxidative stress remains unknown. Therefore, the present study assessed the possible beneficial effects of pterosin A on cell survival and ROS production in insulin-secreting cells subjected to oxidative stress or lipotoxicity. In this study, pterosin A effectively reduced the ROS-induced cell damage in the insulin-secreting cells through the AMPK signaling pathway. Reportedly, pancreatic abnormal glucose metabolism and long-term treatment with FFA can cause defects in mitochondrial function and gradual increase of ROS production, which leads to β-cell dysfunction [16,17,18]. We observed that pterosin A could not reverse the ROS-reduced cell viability but could reduce ROS production. Additional studies focused on detecting the activity of antioxidant enzymes under pterosin A treatment may be required to confirm this indication.

In the present study, pterosin A protected cells against oxidative stress or lipotoxicity-induced damage through AMPK activation. Cotreatment with Compound C inhibited the AMPK activation and eliminated the protective effects of pterosin A on cell viability, with consequent cell injury induced by palmitate or H_2_O_2_. AMPK activation exhibited positive effects on the functional impairment and cell mass of β-cells because of glucotoxicity [13]. Tuberous sclerosis complex 2 (TSC2), downstream of AMPK, can protect against cell death through various signal pathways that regulate cell size, translation, and apoptosis in adverse growth environments [19]. In addition, AMPK activity may be useful to promote the physiological functions of β-cells. Therefore, the protective effects of pterosin A against oxidative damage through AMPK activation presented in our preliminary data may be explained by the aforementioned mechanisms; however, further research is required to confirm these findings.

As described previously, pterosin A is a major compound of pterosins that has antidiabetic and protective effects against β-cell damage. Therefore, pterosin A may be used as a lead compound in the development of drugs for type 2 diabetes. However, the impaired glucose transport in skeletal muscles observed in patients with type 2 diabetes was considered as a major factor responsible for reduced overall glucose uptake in the body [20]. Both insulin stimulation [21] and AMPK activation [22] enhance glucose uptake. In addition, AMPK activation is insulin independent. Moreover, a previous study demonstrated that pterosin A increased the glucose uptake in skeletal muscle cells [4]. In the present study, we screened other pterosin-type compounds to determine whether these pterosins analogs promoted glucose uptake as well, and these pterosins exhibited the same effects in the glucose uptake assays. These findings indicate that pterosins influence various biological processes.

Only few studies have investigated the ptaquiloside content in the products of milk, soil, and groundwater [23] but never the pterosin detection methods. LC-MS-MS is a powerful technique with extremely high sensitivity and selectivity and is thus useful in various applications. In our previous study, we investigated the concentrations of pterosins A, I, and Z present in various fern samples collected from *H. punctata*, *C. thalictroide*, and *P. revolutum* which revealed the same effects in glucose uptake assays. Therefore, the present study is the first to establish an LC–MS–MS method to determine three compounds: pteroisns A, I, and Z. In addition, the present study proposed a method for pterosin detection that presented a clear separation on chromatograms, indicating that this method may be useful to determine the pterosin content in ferns in Taiwan.

## 4. Experimental Section

### 4.1. Chemicals and Reagents

RPMI-1640 and Dulbecco’s modified Eagle’s medium (DMEM) were purchased from Gibco-BRL-Life Technologies (Grand Island, NY, USA); fetal bovine serum from Thermo Scientific (South Logan, UT, USA); (3-(4,5-dimethylthiazol-2-yl)-2,5-diphenyltetrazoliumbromide) MTT, H_2_O_2_, nitrotetrazolium blue chloride (NBT), and 2',7'-dichlorofluorescein diacetate (DCFH-DA) from Sigma Chemical Company (St. Louis, MO, USA); 6-[4-[2-(1-Piperidinyl)ethoxy]phenyl]-3-(4-pyridinyl)-pyrazolo[1,5-a]pyrimidine dihydrochloride (Compound **C**) from Tocris Bioscience (Bristol, UK); antibodies for phospho-AMPK-α (Thr172) and total AMPK from Cell Signaling Technology (Beverly, MA, USA); and horseradish peroxidase-conjugated antirabbit secondary antibody from Jackson (West Grove, PA, USA). The solvents used for column chromatography, including methanol, *i*-PrOH, *n*-BuOH, dichloromethane, chloroform, *n*-hexane, ethyl acetate, acetonitrile, acetone, and formic acid, were purchased from Merck (Darmstadt, Germany).

### 4.2. General Experimental Procedures

The optical rotations were measured using a JASCO P-2000 polarimeter. Infrared (IR) spectra were measured using an Avatar-320-FT-IR spectrometer. In addition, 1D and 2D NMR spectra were obtained by using a Bruker AM-500 (500 MHz) FT-NMR spectrometer with tetramethylsilane as an internal standard. A VG Platform Electrospray mass spectrometer was used for high-resolution electrospray ionization mass spectrometry (HR-ESI-MS). Column chromatography involved the use of Diaion HP 20 (100–200 mesh, Mitsubishi Chemical Industries, Tokyo, Japan), MCI-gel CHP 20P (75–150 μm, Mitsubishi Chemical Industries, Japan), and Cosmosil C18-OPN (75 μm, Nacalai Tesque, Kyoto, Japan). Thin-layer chromatography involved silica gel plates (70–230 mesh, Merck), in which a 10% sulfuric acid solution was used as a visualizing agent during heating.

### 4.3. Plant Material

*H. punctata*, *C. thalictroides*, *N. multiflora*, and *P. revolutum* were collected from Hehuan Mountain, Sun Lake, Jinquashi, and Siyuan Wind Gap, Taiwan, respectively, and were identified by Chen-Meng Kuo (Institute of Ecology and Evolutionary Biology, National Taiwan University, Taiwan). Voucher specimens were deposited at the Department of Medicinal Chemistry, College of Pharmacy, Taipei Medical University.

### 4.4. Extraction and Isolation

(1) *C. thalictroides* (L.) Brongn: Fresh whole plants (50 kg) were extracted three times with MeOH at room temperature. The MeOH extract (490 g) was partitioned between with *n*-hexane/H_2_O and EtOAc/H_2_O. The EtOAc fraction (265.7 g) was chromatographed on a Sephadex LH-20 with 95% EtOH to yield four fractions (CT1–4). The fraction CT1 (30 g) was further applied to MCI gels with an H_2_O-MeOH gradient to yield caffeic acid methyl ester [24] (15, 11.7 mg), quercetin 3-*O*-β-d-glucopyranoside [25] (**22**, 174.2 mg), and kaempherol 3-*O*-β-d-glucopyranoside [25] (**23**, 356.9 mg). The CT2 (20 g) fraction was chromatographed on an MCI-gel CHP 20P with an H_2_O-MeOH gradient to produce CT2.1–2.5; subsequently, each subfraction was further purified on silica gel with a CH_2_Cl_2_-MeOH gradient and a reverse-C_18_ silica gel column with an H_2_O–MeOH gradient to yield *p*-coumaric acid [24] (**16**, 23.8 mg) and *p*-coumaric acid methyl ester [24] (**17**, 7.5 mg). The CT3 (20 g) fraction was chromatographed on silica gel with a CH_2_Cl_2_–MeOH gradient to produce pterosin A [26] (**6**, 6 mg) and pterosin Z [27] (**7**, 3.5 mg). The CT4 (30 g) fraction was repeatedly chromatographed on a Sephadex LH-20 with an H_2_O–MeOH gradient to produce CT4.1–4.4; subsequently, each subfraction was further purified on silica gel with a CH_2_Cl_2_–MeOH gradient to yield Compound **1** (3.6 mg), Compound **2** (2 mg), Compound **3** (3 mg), pterosin D 3-*O*-β-d-glucopyranoside [27] (**4**, 38 mg), pteroside Z [28] (**5**, 14 mg), and 6-*O*-*p*-coumaroyl-d-glucopyranoside [29] (**18**, 3.5 mg).

(2) *H. punctata* (Thumb.) Mett: Fresh whole plants (20 kg) were extracted three times with MeOH at room temperature. The methanolic extract (1.2 kg) was evaporated and partitioned using *n*-hexane/H_2_O to yield *n*-hexane (350 g) and water fractions. The water fraction was further partitioned using EtOAc/H_2_O to obtain EtOAc (230 g) and water fractions (640 g). The EtOAc fraction was chromatographed on a Sephadex LH-20 with an H_2_O–MeOH gradient to yield HP factions 1 to 3. The HP-2 (35.6 g) fraction was subfractionated to HP2.1–2.3 on MCI gels with an H_2_O–MeOH gradient. The HP2.2 fractions were purified using a silica gel column with *n*-hexane–EtOAc (3:1 to 2:1) to yield pterosin A [26] (**6**, 4 g), pterosin Z [27] (**7**, 2.3 g), pterosin D [27] (**8**, 50 mg), and pterosin I [27] (**9**, 187 mg). The fraction HP1 (42 g) was chromatographed on a MCI-gel CHP 20P with an H_2_O–MeOH gradient to produce subfractions HP1.1–1.5; each subfraction was then further purified on silica gel with a CH_2_Cl_2_–MeOH gradient, Sephadex LH-20 with acetone, and a reverse-C_18_ silica gel column with an H_2_O–MeOH gradient to yield quercetin [30] (**21**, 1.2 g), quercetin 3-*O*-β-d-glucopyranoside [25] (**22**, 394 mg), and kaempherol 3-*O*-β-d-glucopyranoside [25] (**23**, 355 mg).

(3) *N. multiflora* (Roxb.) Jarret ex Morton: Fresh whole plants (20 kg) were extracted three times with MeOH and then concentrated to a residue (460.8 g) under vacuum at 40 °C, dissolved in H_2_O, and partitioned between *n*-hexane/H_2_O, EtOAc/H_2_O, and CH_2_Cl_2_/H_2_O to produce four layers. The concentrated EtOAc extract (35.5 g) was subjected to column chromatography on a Sephadex LH20 column and silica gel eluted with a CH_2_Cl_2_–MeOH gradient to produce kaempherol 3-*O*-β-d-glucopyranoside [25] (**23**, 23.6 mg), matairesinoside [31] (**29**, 15.8 mg), shikimic acid [32] (**19**, 95.5 mg), ethyl shikimate [33] (**20**, 25 mg), and ethyl β-d-fructopyranoside [34] (**30**, 16.8 mg). Subsequently, the CH_2_Cl_2_ fraction (22.1 g) was chromatographed on Sephadex LH-20 with 95% EtOH to yield three fractions, which were further purified on silica gel and *n*-hexane-EtOAc along with an MCI-gel column with an H_2_O–MeOH gradient to yield arctigenin [35] (**24**, 412.1 mg) and arctiin [35] (**28**, 88 mg).

(4) *P. revolutum* (BI.) Nakai: Fresh whole plants (50 kg) were extracted with MeOH at room temperature; after evaporation of the organic solvent, the extract was subjected to Celite CC sequential elute with *n*-hexane, CH_2_Cl_2_, and MeOH to produce three fractions. The CH_2_Cl_2_ extract (151.1 g) underwent column chromatography on the MCI gel eluted with an H_2_O–MeOH gradient to produce three fractions. The PR1 (12.4 g) fraction was purified on silica gel (CH_2_Cl_2_/MeOH 14:1) to produce three subfractions PR1–3. The PR1.1 (8.7 g) fraction was applied on silica gel (*n*-hexane/EtOAc 1:2) and reverse-C_18_ silica gel (CH_3_CN/H_2_O 20:80–30:70) to produce pterosin A [26] (**6**, 238 mg), (2*R*,3*R*)-pterosin L [12] (**10**, 179 mg), and pterosin G [36] (**11**, 73 mg). The PR1.2 (1.5 g) fraction was then chromatographed on silica gel (*n*-hexane/EtOAc 1:2) and a reverse-C_18_ silica gel (CH_3_CN/H_2_O 10:90) to produce 2-hydroxypterosin C [27] (**12**, 4.3 mg). The PR2 (4.8 g) fraction was purified on silica gel (*n*-hexane/EtOAc 1:2) to produce eight fractions. The PR2.5 fraction was further purified on silica gel with hexane–EtOAc to yield (2*S*,3*S*)-pterosin C [36] (**13**, 162 mg), (2*R*,3*S*)-pterosin C [37] (**14**, 13 mg), balanophonin [38] (**25**, 45.3 mg), pinoresinol [39] (**26**, 251 mg), and lariciresinol [40] (**27**, 7 mg).

13-Chloro-spelosin 3-*O*-*β*-d-glucopyranoside (**1**): colorless oil; [α]_D_^25^ + 9.08 (*c* = 1.0, MeOH); IR (KBr) *v*_max_ : 3387, 1697, and 1598 cm^−^^1^; UV (MeOH) λ_max_: 211, 227, and 311 nm; ^1^H- and ^13^C-NMR spectra (Table 1); HR-ESI-MS *m*/*z*: 445.1631 [M + H]^+^ (calcd. for C_21_H_29_ClO_8_, 445.1629).

(3*R*)-pterosin D 3-*O*-β-d-(3'-*p*-coumaroyl)-glucopyranoside (**2**): colorless oil; [α]_D_^25^ − 11.04 (*c* = 1.0, MeOH); IR (KBr) *v*_max_: 3408, 2962, 1697, and 1603 cm^−^^1^; UV (MeOH) λ_max_: 218, 260, and 310 nm; ^1^H- and ^13^C-NMR spectra (Table 1); HR-ESI-MS *m*/*z*: 556.2312 [M + Na]^+^ (calcd. for C_30_H_36_O_11_Na, 556.2308).

(2*R*,3*R*)-pterosin L 3-*O*-β-d-(3'-*p*-coumaroyl)-glucopyranoside (**3**): colorless oil; [α]_D_^25^ − 26.0 (*c* = 1.0, MeOH); UV (MeOH) λ_max_: 218, 260, and 310 nm; IR (KBr) *v*_max_: 3419, 2931, 1695, and 1605 cm^−^^1^; ^1^H- and ^13^C-NMR spectra (Table 1); HR-ESI-MS *m*/*z*: 572.2263 [M + Na]^+^ (calcd. for C_30_H_36_O_11_Na, 572.2258).

Acid hydrolysis of compounds **1**–**3**. Compounds **1**–**3** (2 mg) were treated with 2 N HCl in aqueous MeOH (2 mL) for 4 h, and the reaction mixture was further extracted with EtOAc. The EtOAc layer was removed *in vacuo* and the residue was passed through the silica gel with eluent of *n*-hexane/EtOAc to yield 13-chloro-spelosin, (3*R*)-pterosin D and (2*R*,3*R*)-pterosin L, respectively. The sugar was analyzed by silica gel TLC [*i*-PrOH–Me_2_CO–H_2_O (5:3:1)] comparison with an authentic sample.

### 4.5. Pterosin Analysis by LC–MS–MS

Three pterosin compounds (pterosins A, I, and Z, 120 μg/mL and internal standard stock solution (piromidic acid, 11.1 μg/mL) were prepared. Separation involved a reverse-phase C18 column (Cosmosil MS-II, 3C18, 4.6 × 100 mm) under gradient elution. The mobile phase comprised a mixed solvent system of acetonitrile/H_2_O/0.25% formic acid (A/B/C) at a 220-nm wavelength. The elution conditions were maintained at 20/60/20 to 80/0/20 (A/B/C) for 0 to 25 min (linear gradient) and 80/0/20 (A/B/C) for 5 min, set at a flow rate of 0.5 mL/min with a split ratio of 1:1 in a photodiode array and a tandem mass spectrophotometer. ESI was used for operating the ion source in the positive mode, which was monitored using multiple reaction monitoring (MRM). The source and desolvation temperatures were set at 120 and 350 °C, respectively. The desolvation gas flow (N_2_) was 600 L/h, and the cone gas flow (N_2_) was 60 L/h. The capillary and cone voltages were 3.0 kV and 80 V, respectively. The collision energies were optimized for each compound. Qualitative analysis was achieved by daughter ion analysis.

### 4.6. Cell Culture

C2C12 myoblast and rat pancreatic insulin-secreting (RINm5F) cells were obtained from the American Type Culture Collection (Rockville, MD, USA). The cells were maintained in DMEM and RPMI-1640 medium at 37 °C in an atmosphere of 5% CO_2_.

### 4.7. Biological Validation

#### 4.7.1. Determination of Glucose Uptake in C2C12 Myocytes

Glucose uptake was determined based on the uptake of the radioactive glucose analogue 2-deoxy-d-[^3^H] glucose (Sigma-Aldrich, St. Louis, MO, USA) as described previously [41]. The C2C12 myocytes were washed with phosphate-buffered saline (PBS) and incubated in serum-free DMEM and then treated with pterosin compounds (1 μM) at 37 °C for 1 h. The glucose uptake was determined by adding 0.5 μCi 2-deoxy-d-[^3^H] glucose for 20 min. The reaction was terminated using ice-cold PBS. After centrifugation, the cells were washed twice with ice-cold PBS to remove extrinsic glucose and lysed with 0.1% SDS; the glucose uptake was then estimated using a scintillation counter.

#### 4.7.2. Measurement of ROS and Cell Viability

ROS levels were determined by NBT analysis as described previously [42]. The cells were seeded in 24-well plates at 2 × 10^5^ cell/well and then treated with pterosin A at various doses and incubated for 18 h. The absorbance was recorded at 630 nm. Cell viability was measured by MTT assay. The RINm5F cells were seeded in 24-well plates at 2 × 10^5^ cell/0.5 mL and grown for 3 days for adherence. Subsequently, 50 μL of MTT solution (1 mg/mL in PBS) were added to each well for 2 h at 37 °C. The medium was aspirated, and 200 μL of DMSO were added. After the formazan product was dissolved, the absorbance at 570 nm was measured using a spectrophotometer.

#### 4.7.3. Immunofluorescence Study

Intracellular oxidation was analyzed using a fluorometric assay with DCFH-DA. DCFH-DA transports across the cell membrane and deacetylates by cellular esterases to nonfluorescent DCFH, which quickly oxidizes to highly fluorescent DCF by ROS [43]. The RINm5F cells (3 × 10^5^ cell/well in 12 wells) were exposed to different treatments for varying durations after adhering for 3 days. In total, 10 μM of DCFH-DA was added with no serum medium for 20 min. The cells were washed two times with PBS and then subjected to DCF fluorescence by using fluorescence microscopy at 488-nm excitation (argon laser) and 515-nm long-pass emission.

#### 4.7.4. Western Blot Analysis

Total cellular proteins were separated by SDS-PAGE and transferred onto polyvinylidene difluoride membranes for immunoblotting. Nonspecific binding was blocked using a blocking buffer containing 5% fat-free milk powder in Tris-buffered saline with 1% Tween-20 for 1 h at room temperature. The lysates were incubated with monoclonal antibodies against phospho-AMPK and total AMPK. The protein expression was determined using an enhanced chemiluminescence kit (Amersham International, Amersham, UK).

### 4.8. Statistical Analysis

The significance of various treatments was determined by one-way analysis of variance. Data were expressed as mean ± SEM. Statistically significant differences were considered at *p* < 0.05.

## 5. Conclusions

This paper reports the isolation of pterosin-type compounds (discovered in three fern species: *H. punctata*, *C. thalictroides*, and *P. revolutum*), that have the same effects on glucose uptake assays as known isolated pterosins. In addition, three new compounds were isolated from the *C. thalictroides* fern. Moreover, the present study is the first to demonstrate that pterosin A has protective effects on insulin secretion in cells against ROS- and palmitate-induced cell damage. We provide information regarding these signals with pterosin-like UV spectra in the chromatographic system, which is vital to determine the pterosin-type constituents in ferns.

## Supplementary Materials

Supplementary materials can be found at http://www.mdpi.com/1422-0067/16/02/2947/s1.

## References

[1] Ho R., Teai T., Bianchini J.-P., Lafont R., Raharivelomanana P., Fernández H., Revilla M.A., Kumar A. (2010). Working with ferns: Issues and applications. Ferns: From Traditional Uses to Pharmaceutical Development, Chemical Identification of Active Principles.

[2] Hikino H., Takahashi T., Arihara S., Takemoto T. (1970). Structure of pteroside B, glycoside of *Pteridium aquilinum* var latiusculum. Chem. Pharm. Bull..

[3] Yoshihira K., Fukuoka M., Kuroyannagi M., Natori S., Umeda M., Morohoshi T., Enomoto M., Saito M. (1978). Chemical and toxicological studies on bracken fern, *Pteridium aquilinum* var. latiusculum. I. Introduction, extraction and fractionation of constituents, and toxicological studies including carcinogenicity tests. Chem. Pharm. Bull. (Tokyo).

[4] Hsu F.L., Huang C.F., Chen Y.W., Yen Y.P., Wu C.T., Uang B.J., Yang R.S., Liu S.H. (2013). Antidiabetic effects of pterosin A, a small-molecular-weight natural product, on diabetic mouse models. Diabetes.

[5] Xiong F.L., Sun X.H., Gan L., Yang X.L., Xu H.B. (2006). Puerarin protects rat pancreatic islets from damage by hydrogen peroxide. Eur. J. Pharmacol..

[6] Kaneto H., Kawamori D., Matsuoka T.-A., Kajimoto Y., Yamasaki Y. (2005). Oxidative stress and pancreatic β-cell dysfunction. Am. J. Ther..

[7] Shimabukuro M., Ohneda M., Lee Y., Unger R.H. (1997). Role of nitric oxide in obesity-induced β cell disease. J. Clin. Investig..

[8] Fonseca S.G., Gromada J., Urano F. (2011). Endoplasmic reticulum stress and pancreatic β-cell death. Trends Endocrinol. Metab..

[9] Rutter G.A., da Silva Xavier G., Leclerc I. (2003). Roles of 5'-AMP-activated protein kinase (AMPK) in mammalian glucose homoeostasis. Biochem. J..

[10] Eto K., Yamashita T., Matsui J., Terauchi Y., Noda M., Kadowaki T. (2002). Genetic manipulations of fatty acid metabolism in β-cells are associated with dysregulated insulin secretion. Diabetes.

[11] Richards S.K., Parton L.E., Leclerc I., Rutter G.A., Smith R.M. (2005). Over-expression of AMP-activated protein kinase impairs pancreatic β-cell function *in vivo*. J. Endocrinol..

[12] Kuraishi T., Murakami T., Taniguchi T., Kobuki Y., Maehashi H., Tanaka N., Saiki Y., Chen C.M. (1985). Chemical and chemotaxonomical studies of ferns. LIV. Pterosin derivatives of the genus *Microlepia* (*Pteridaceae*). Chem. Pharm. Bull..

[13] Nyblom H.K., Sargsyan E., Bergsten P. (2008). AMP-activated protein kinase agonist dose dependently improves function and reduces apoptosis in glucotoxic β-cells without changing triglyceride levels. J. Mol. Endocrinol..

[14] Lin N., Chen H., Zhang H., Wan X., Su Q. (2012). Mitochondrial reactive oxygen species (ROS) inhibition ameliorates palmitate-induced INS-1 β cell death. Endocrine.

[15] Banerjee J., Datta G., Duita C.P., Som U.K. (1988). Chemical constituents of *Nephrolepis tuberosa*. J. Indian Chem. Soc..

[16] Carlsson C., Håkan Borg L.A., Welsh N. (1999). Sodium palmitate induces partial mitochondrial uncoupling and reactive oxygen species in rat pancreatic islets *in vitro*. Endocrinology.

[17] Evans J.L., Goldfine I.D., Maddux B.A., Grodsky G.M. (2003). Are oxidative stress-activated signaling pathways mediators of insulin resistance and β-cell dysfunction?. Diabetes.

[18] Wang X., Li H., de Leo D., Guo W., Koshkin V., Fantus I.G., Giacca A., Chan C.B., Der S., Wheeler M.B. (2004). Gene and protein kinase expression profiling of reactive oxygen species-associated lipotoxicity in the pancreatic β-cell line MIN6. Diabetes.

[19] Inoki K., Zhu T., Guan K.-L. (2003). TSC2 mediates cellular energy response to control cell growth and survival. Cell.

[20] Zierath J.R., He L., Guma A., Odegoard Wahlstrom E., Klip A., Wallberg-Henriksson H. (1996). Insulin action on glucose transport and plasma membrane GLUT4 content in skeletal muscle from patients with NIDDM. Diabetologia.

[21] Wang Q., Somwar R., Bilan P.J., Liu Z., Jin J., Woodgett J.R., Klip A. (1999). Protein kinase B/Akt participates in GLUT4 translocation by insulin in L6 myoblasts. Mol. Cell Biol..

[22] Czech M.P., Corvera S. (1999). Signaling mechanisms that regulate glucose transport. J. Biol. Chem..

[23] Jensen P.H., Jacobsen O.S., Hansen H.C., Juhler R.K. (2008). Quantification of ptaquiloside and pterosin B in soil and groundwater using liquid chromatography-tandem mass spectrometry (LC–MS/MS). J. Agric. Food Chem..

[24] Kelley C.J., Harruff R.C., Carmack M. (1976). Polyphenolic acids of Lithospermum ruderale. II. Carbon-13 nuclear magnetic resonance of lithospermic and rosmarinic acids. J. Org. Chem..

[25] Zhang H.L., Nagatsu A., Okuyama H., Mizukami H., Sakakibara J. (1998). Sesquiterpene glycosides from cotton oil cake. Phytochemistry.

[26] Hikino H., Takahashi T., Takemoto T. (1972). Structure of pteroside A and C, glycosides of *Pteridium aquilinum* var latiusculum. Chem. Pharm. Bull..

[27] Tanaka N., Satake T., Takahashi A., Mochizuki M., Murakami T., Saiki Y., Yang J.Z., Chen C.M. (1982). Chemical and chemotaxonomical studies of ferns. XXXIX. Chemical studies on the constituents of *Pteris bella* Tagawa and *Pteridium aquilinum* subsp. wightianum (Wall) Shich. Chem. Pharm. Bull..

[28] Hikino H., Takahashi T., Takemoto T. (1971). Structure of pterosides Z and D, glycosides of *Pteridium aquilinum* var latiusculum. Chem. Pharm. Bull..

[29] Shimomura H., Sashida Y., Adachi T. (1988). Phenylpropanoid glucose esters from *Prunus buergeriana*. Phytochemistry.

[30] Markham K.R., Ternai B., Stanley R., Geiger H., Mabry T.J. (1978). Carbon-13 NMR studies of flavonoids. III. Naturally occurring flavonoid glycosides and their acylated derivatives. Tetrahedron.

[31] Abe F., Yamauchi T. (1986). Lignans from *Trachelospermum asiaticum* (Tracheolospermum. II). Chem. Pharm. Bull..

[32] Talapatra B., Das A.K., Talapatra S.K. (1988). On the chemistry of Indian Orchidaceae plants. Part V. Defuscin, a new phenolic ester from *Dendrobium fuscescens*: Conformation of shikimic acid. Phytochemistry.

[33] Xie C., Li Z., Qu J., Sun B., Lou H. (2007). Chemical constituents of two liverworts *Dumortiera hirsute* and *Pallavicinia ambigua*. Chin. Pharm. J..

[34] Kuang H.X., Kasai R., Ohtani K., Liu Z.S., Yuan C.S., Tanaka O. (1989). Chemical constituents of pericarps of *Rosa davurica* Pall., a traditional Chinese medicine. Chem. Pharm. Bull..

[35] Rahman M.M.A., Dewick P.M., Jackson D.E., Lucas J.A. (1990). Lignans of *Forsythia intermedia*. Phytochemistry.

[36] Yoshihira K., Fukuoka M., Kuroyanagi M., Natori S. (1972). Further characterization of 1-indanone derivatives from bracken, *Pteridium aquilinum* var latiusculum. Chem. Pharm. Bull..

[37] Tanaka N., Murakami T., Saiki Y., Chen C.M., Gomez P.L.D. (1981). Chemical and chemotaxonomical studies of ferns. XXXVII. Chemical studies on the constituents of Costa Rican ferns. 2. Chem. Pharm. Bull..

[38] Warashina T., Nagatani Y., Noro T. (2005). Further constituents from the bark of *Tabebuia impetiginosa*. Phytochemistry.

[39] Nishibe S., Tsukamoto H., Hisada S. (1984). Effects of *O*-methylation and *O*-glucosylation on carbon-13 nuclear magnetic resonance chemical shifts of matairesinol, (+)-pinoresinol and (+)-epipinoresinol. Chem. Pharm. Bull..

[40] El Gamal A.A., Takeya K., Itokawa H., Halim A.F., Amer M.M., Saad H.E.A. (1997). Lignan *bis*-glucosides from *Galium sinaicum*. Phytochemistry.

[41] Park C.E., Kim M.J., Lee J.H., Min B.I., Bae H., Choe W., Kim S.S., Ha J. (2007). Resveratrol stimulates glucose transport in C2C12 myotubes by activating AMP-activated protein kinase. Exp. Mol. Med..

[42] Choi H.S., Kim J.W., Cha Y.N., Kim C. (2006). A quantitative nitroblue tetrazolium assay for determining intracellular superoxide anion production in phagocytic cells. J. Immunoass. Immunochem..

[43] Rosenkranz A.R., Schmaldienst S., Stuhlmeier K.M., Chen W., Knapp W., Zlabinger G.J. (1992). A microplate assay for the detection of oxidative products using 2',7'-dichlorofluorescein-diacetate. J. Immunol. Methods.

